# Supplementary material for: Single-cell and spatial detection of senescent cells using DeepScence
Source: Cell Genom. 2025 Oct 7;5(12):101035. doi: 10.1016/j.xgen.2025.101035 (PMC12802646; doi:10.1016/j.xgen.2025.101035)
Supplement: Document S1. Figures S1–S4 [file mmc1.pdf]

Cell Genomics, Volume *5*

Supplemental information

Single-cell and spatial detection of senescent cells using DeepScence

Yilong Qu, Beijie Ji, Runze Dong, Liangcai Gu, Cliburn Chan, Jichun Xie, Carolyn Glass, Xiao-Fan Wang, Andrew B. Nixon, and Zhicheng Ji

SUPPLEMENTAL FIGURES

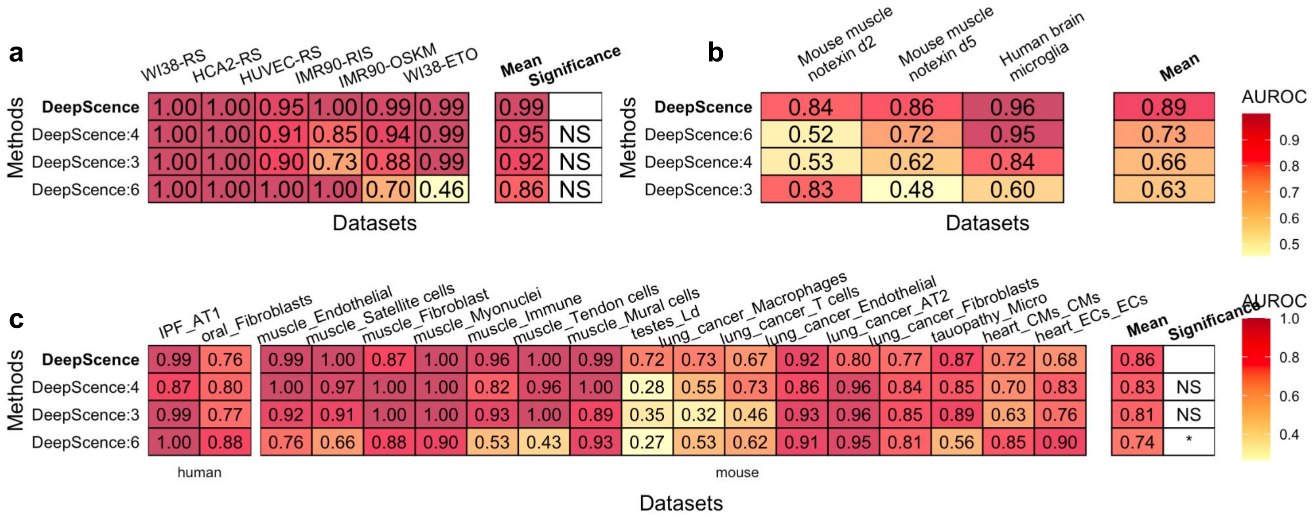

Figure S1: DeepScence performance using different CoreScence subset, related to Figure 1, Figure 3, and Figure 4

AUROCs for DeepScence using different thresholds for constructing CoreScence across *in vitro* scRNA-seq datasets[1–6] (**a**), ST datasets [7, 8] (**b**), and *in vivo* scRNA-seq datasets [7, 9–14] (**c**). Methods are ordered in decreasing order of average AUROC. Paired t-tests were conducted to compare the performance between default DeepScence and its variants. "*" indicates $0.01 <$ p-value < 0.05, and "NS" indicates not significant (p-value > 0.05).

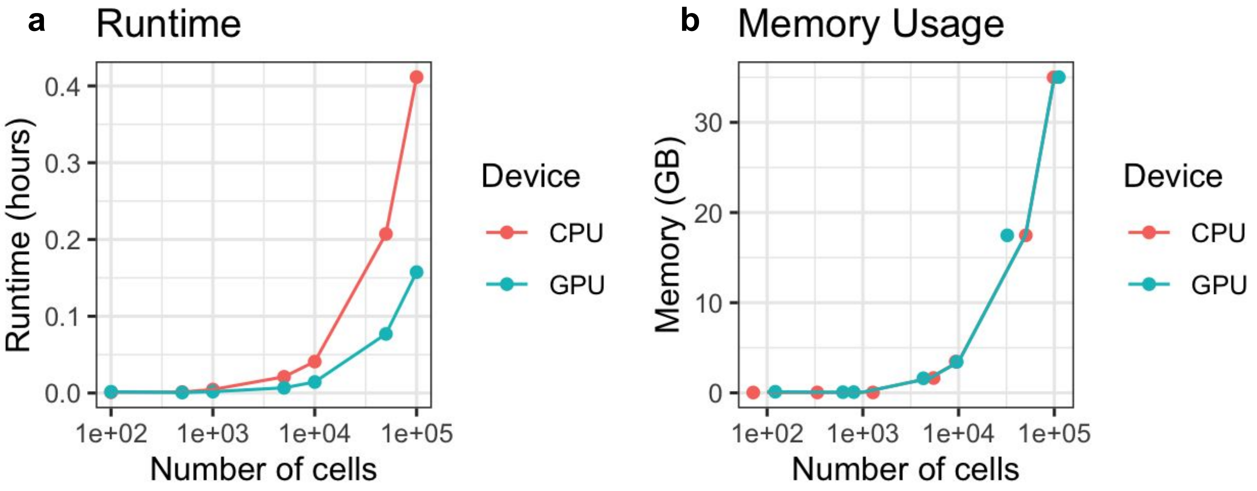

Figure S2: Computational efficiency of DeepScence, related to Figure 3 and STAR Methods

Runtime (**a**) and peak memory usage (**b**) for running DeepScence with different numbers of cells in the input data.

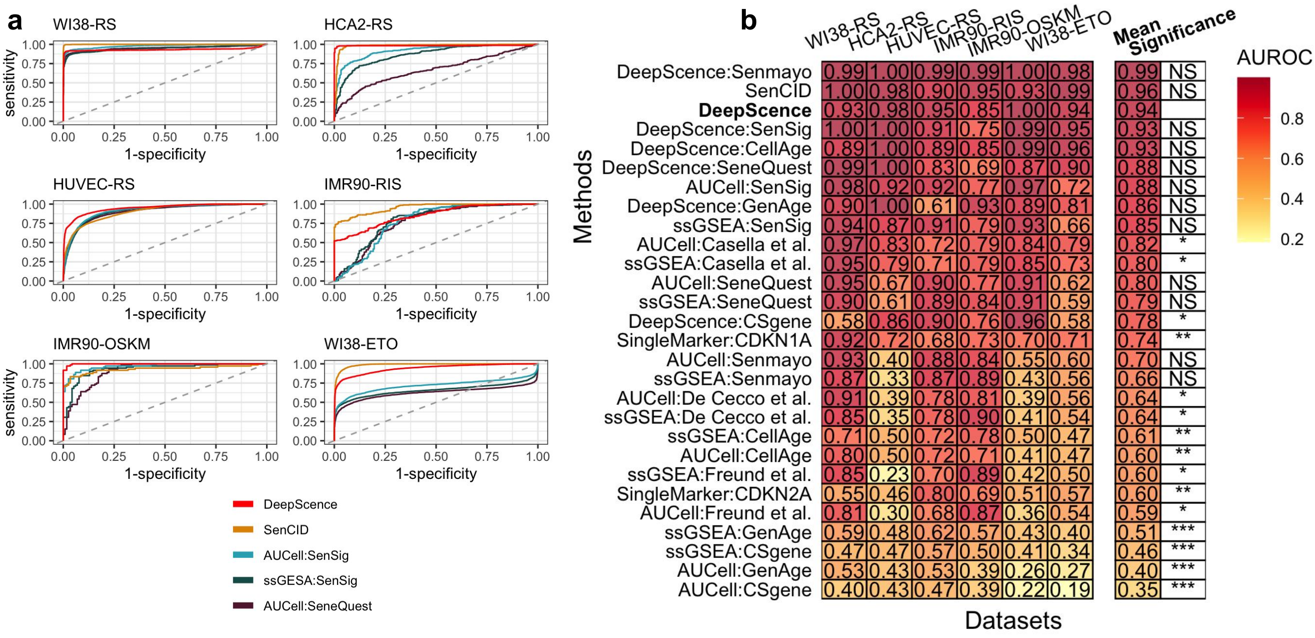

Figure S3: DeepScence performance *in vitro* using a standard preprocessing pipeline, related to Figure 3

a, AUC curves for the top 5 performing methods among *in vitro* datasets [1–6] under standard data preprocessing pipeline. **b,** AUROCs for all methods among *in vitro* datasets under standard data preprocessing pipeline. Methods are ordered in decreasing order by average AUROCs. Paired t-test was conducted to compare the performance between DeepScence and each of the other methods. "***" indicates p-value < 0.001, "**" indicates $0.001 <$ p-value < 0.01, "*" indicates $0.01 <$ p-value < 0.05, and "NS" indicates not significant (p-value > 0.05).

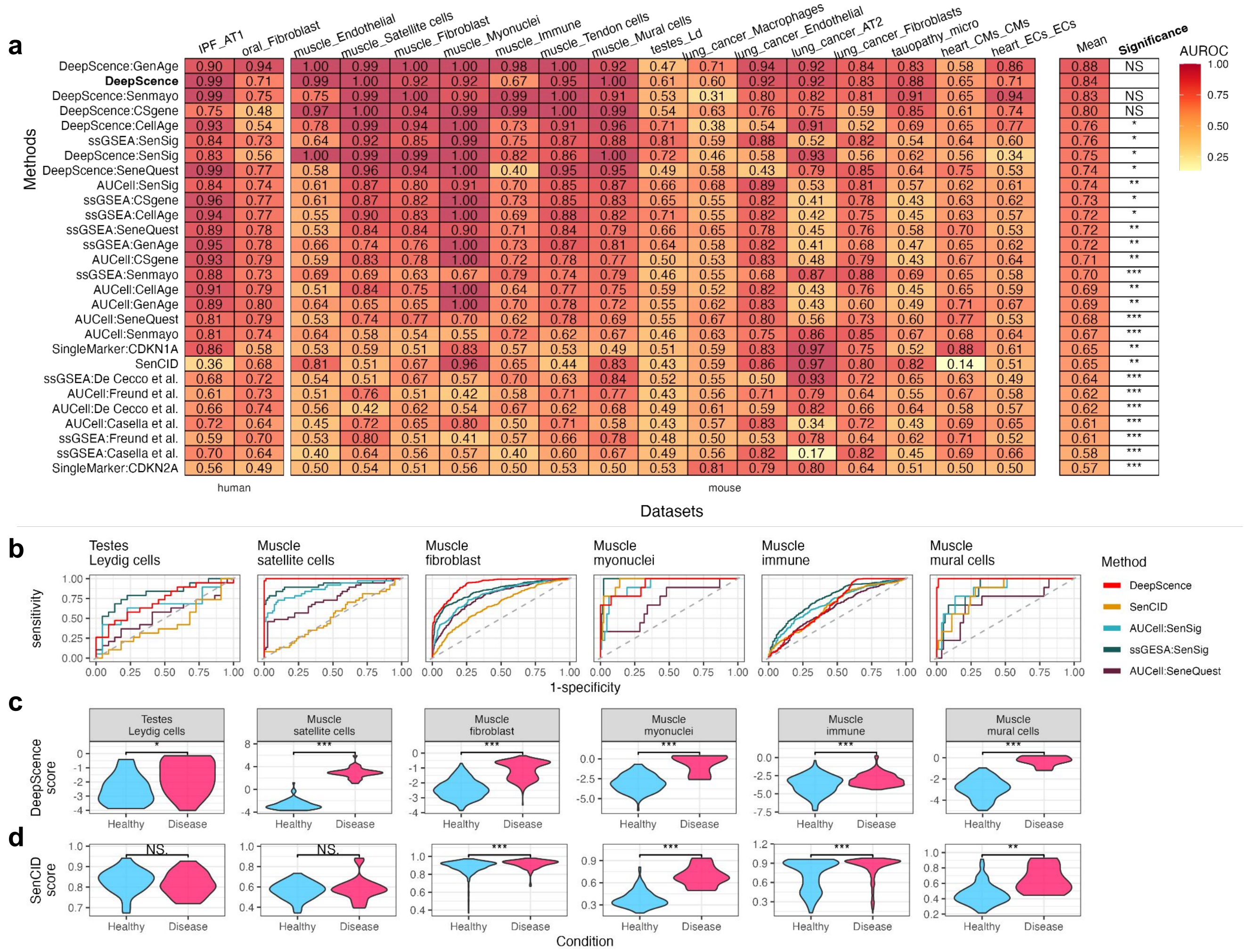

Figure S4: DeepScence performance *in vivo* using a standard preprocessing pipeline, related to Figure 4

a, AUROCs for all methods across *in vivo* datasets [7, 9–14] under standard preprocessing pipeline. Methods are ordered in decreasing order by average AUROCs. Paired t-test was conducted to compare the performance between DeepScence and each of the other methods. "***" indicates p-value < 0.001, "**" indicates $0.001 <$ p-value < 0.01, "*" indicates $0.01 <$ p-value < 0.05, and "NS" indicates not significant (p-value > 0.05). **b,** ROC curves for six example cell types in *in vivo* datasets under standard preprocessing, showing methods with top performance in *in vitro* datasets. **c,** Distribution of SenCID and DeepScence scores under standard preprocessing, comparing cells from healthy and diseased conditions. Wilcoxon test was conducted to compare the two distributions in each case. '*' indicates p-value between 0.01 and 0.05, '**' indicates p-value between 0.001 and 0.01, and '***' indicates p-value < 0.001.

References

1. M, C., H, Y., I, S., Tm, M., Ry, W., A, I., Jj, O., J, G., Ljg, C., T, V., A, F., C, K., Bd, B., Fe, M., Dr, K., M, R., Rl, C., Ad, L., D, B., and Dg, H. (2022). Novel insights from a multiomics dissection of the Hayflick limit. eLife *11*. Publisher: Elife.

2. Tang, H., Geng, A., Zhang, T., Wang, C., Jiang, Y., and Mao, Z. (2019). Single senescent cell sequencing reveals heterogeneity in senescent cells induced by telomere erosion. Protein & Cell *10*, 370–375.

3. A, Z., M, N., K, S., Jp, M., Ca, B., H, G., O, D., C, B., J, A., N, J., T, G., L, B., J, F., M, K., Eg, G., Ig, C., Rt, U., W, W., V, R., P, N., D, M., K, R., and A, P. (2018). HMGB2 Loss upon Senescence Entry Disrupts Genomic Organization and Induces CTCF Clustering across Cell Types. Molecular cell *70*. Publisher: Mol Cell.

4. Teo, Y.V., Rattanavirotkul, N., Olova, N., Salzano, A., Quintanilla, A., Tarrats, N., Kiourtis, C., Müller, M., Green, A.R., Adams, P.D., Acosta, J.C., Bird, T.G., Kirschner, K., Neretti, N., and Chandra, T. (2019). Notch Signaling Mediates Secondary Senescence. Cell Reports *27*, 997–1007.e5.

5. Aarts, M., Georgilis, A., Beniazza, M., Beolchi, P., Banito, A., Carroll, T., Kulisic, M., Kaemena, D.F., Dharmalingam, G., Martin, N., Reik, W., Zuber, J., Kaji, K., Chandra, T., and Gil, J. (2017). Coupling shRNA screens with single-cell RNA-seq identifies a dual role for mTOR in reprogramming-induced senescence. Genes & Development *31*, 2085–2098.

6. Wechter, N., Rossi, M., Anerillas, C., Tsitsipatis, D., Piao, Y., Fan, J., Martindale, J.L., De, S., Mazan-Mamczarz, K., and Gorospe, M. (2023). Single-cell transcriptomic analysis uncovers diverse and dynamic senescent cell populations. Aging *15*, 2824–2851.

7. Young, L.V., Wakelin, G., Cameron, A.W.R., Springer, S.A., Ross, J.P., Wolters, G., Murphy, J.P., Arsenault, M.G., Ng, S., Collao, N., De Lisio, M., Ljubicic, V., and Johnston, A.P.W. (2022). Muscle injury induces a transient senescence-like state that is required for myofiber growth during muscle regeneration. FASEB journal: official publication of the Federation of American Societies for Experimental Biology *36*, e22587.

8. McKellar, D.W., Walter, L.D., Song, L.T., Mantri, M., Wang, M.F.Z., De Vlaminck, I., and Cosgrove, B.D. (2021). Large-scale integration of single-cell transcriptomic data captures transitional progenitor states in mouse skeletal muscle regeneration. Communications Biology *4*, 1280.

9. Li, Y., Mi, P., Wu, J., Tang, Y., Liu, X., Cheng, J., Huang, Y., Qin, W., Cheng, C.Y., and Sun, F. (2021). High Throughput scRNA-Seq Provides Insights Into Leydig Cell Senescence Induced by Experimental Autoimmune Orchitis: A Prominent Role of Interstitial Fibrosis and Complement Activation. Frontiers in Immunology *12*, 771373.

10. Sui, J., Boatz, J.C., Shi, J., Hu, Q., Li, X., Zhang, Y., Königshoff, M., and Kliment, C.R. (2023). Loss of ANT1 Increases Fibrosis and Epithelial Cell Senescence in Idiopathic Pulmonary Fibrosis. American Journal of Respiratory Cell and Molecular Biology *69*, 556–569. Publisher: American Thoracic Society - AJRCMB.

11. Guo, S., Fu, L., Yin, C., Shao, W., Sun, Q., Chen, L., Xia, T., Wang, M., and Xia, H. (2024). ROS-Induced Gingival Fibroblast Senescence: Implications in Exacerbating Inflammatory Responses in Periodontal Disease. Inflammation.

12. Ng, P.Y., Zhang, C., Li, H., and Baker, D.J. (2023). Senescent Microglia Represent a Subset of Disease-Associated Microglia in P301S Mice. Journal of Alzheimer's disease: JAD *95*, 493–507.

13. Katoh, M., Nomura, S., Yamada, S., Ito, M., Hayashi, H., Katagiri, M., Heryed, T., Fujiwara, T., Takeda, N., Nishida, M., Sugaya, M., Kato, M., Osawa, T., Abe, H., Sakurai, Y., Ko, T., Fujita, K., Zhang, B., Hatsuse, S., Yamada, T., Inoue, S., Dai, Z., Kubota, M., Sawami, K., Ono, M., Morita, H., Kubota, Y., Mizuno, S., Takahashi, S., Nakanishi, M., Ushiku, T., Nakagami, H., Aburatani, H., and Komuro, I. (2024). Vaccine Therapy for Heart Failure Targeting the Inflammatory Cytokine Igfbp7. Circulation *150*, 374–389. Publisher: American Heart Association.

14. Haston, S., Gonzalez-Gualda, E., Morsli, S., Ge, J., Reen, V., Calderwood, A., Moutsopoulos, I., Panousopoulos, L., Deletic, P., Carreno, G., Guiho, R., Manshaei, S., Gonzalez-Meljem, J.M., Lim, H.Y., Simpson, D.J., Birch, J., Pallikonda, H.A., Chandra, T., Macias, D., Doherty, G.J., Rassl, D.M., Rintoul, R.C., Signore, M., Mohorianu, I., Akbar, A.N., Gil, J., Muñoz-Espín, D., and Martinez-Barbera, J.P. (2023). Clearance of senescent macrophages ameliorates tumorigenesis in KRAS-driven lung cancer. Cancer Cell *41*, 1242–1260.e6. Publisher: Elsevier.
